# Optimization, characterization and biosafety of oregano, rosemary and mint oil mixture against *Penicillium digitatum* in citrus using L-optimal mixture design

**DOI:** 10.1186/s13568-024-01806-0

**Published:** 2025-01-27

**Authors:** Rahaf Khaled, Sara Mohamed, Amira Mohamed, Aya Khairy, Hesham Elhariry, Ashraf Bakry, Neima K. Elsenosy, Naglaa Ebeed, Salem S. Salem, Thanaa ElNoby, Samah H. Abu-Hussien

**Affiliations:** 1 Biotechnology Program, New Programs Administration, Faculty of Agriculture, Ain Shams University, P.O. Box 68, Cairo, 11241 Egypt; 2 Department of Food Science, Faculty of Agriculture, Ain Shams University, P.O. Box 68, Cairo, 11241 Egypt; 3 Department of Genetics, Faculty of Agriculture, Ain Shams University, P.O. Box 68, Cairo, 11241 Egypt; 4 Department of Botany and Microbiology, Faculty of Science, Al-Azhar University, Nasr City, Cairo Egypt; 5 Department of Agricultural Economics, Faculty of Agriculture, Ain Shams University, P.O. Box 68, Cairo, 11241 Egypt; 6 Department of Agricultural Microbiology, Faculty of Agriculture, Ain Shams University, P.O. Box 68, Cairo, 11241 Egypt

**Keywords:** Essential oils, *Penicillium digitatum*, Antifungal activity, Antioxidant potential, Postharvest disease management

## Abstract

The increasing demand for natural alternatives to synthetic fungicides has prompted research into natural products like essential oils for postharvest disease management. This study investigated the antifungal, antioxidant, cytotoxic, and genotoxic potential of essential oil mixtures derived from oregano, rosemary, and mint against *Penicillium digitatum*, the predominant fungal pathogen causing green mold in orange fruits. *P. digitatum* NPAGRASU 2024 was isolated and identified as the most abundant species (60.2%) from infected oranges. It was deposited in GenBank with gene accession number PP930644 and deposited in MIRCEN culture collection as EMCC 358874. The antimicrobial activity of individual essential oils was evaluated, with oregano exhibiting the highest antifungal activity (inhibition zone diameter of 4.2 cm) against *P. digitatum*. L-optimal mixture design of response surface methodology (RSM) optimization revealed a highly effective mixture (Run 8) comprising 46.26% oregano and 53.74% rosemary, with a 99.65% actual growth reduction. The oregano oil demonstrated potent antioxidant activity, reaching approximately 75% DPPH radical scavenging at 3.125 mg/mL. Cytotoxicity assessment using the MTT assay showed morphological changes and reduced cell viability in liver cells treated with the PEOs mixture at 300 µg/mL. However, the optimized mixture did not induce significant chromosomal aberrations compared to the control, suggesting minimal genotoxic effects. In vivo, evaluation on oranges revealed 60% inhibition of green mold by the 1% (v/v) optimized mixture for 7 days. Histological analysis indicated low toxicity to the liver at the highest tested concentration (1% mixture). GC-MS analysis identified major compounds like cavarcol, caryophyllene, eucalyptol, phenols and levomenthol in oregano oil, contributing to its bioactivities. This study demonstrates the potential of optimized essential oil mixtures as effective and eco-friendly alternatives for postharvest disease control.

**Supplementary Information:**

The online version contains supplementary material available at 10.1186/s13568-024-01806-0.

## Key points

- Oregano-rosemary mix shows 99.65% growth reduction against *P. digitatum* in citrus green mold.
- Essential oil mixture exhibits antifungal and antioxidant properties with minimal genotoxicity.
- Invivo studies revealed that 1% optimized mixture inhibits 60% of green mold on oranges for 7 days.

**Supplementary Information:**

The online version contains supplementary material available at 10.1186/s13568-024-01806-0.

## Introduction

Fungal contamination is a major concern in the agricultural and food industries, particularly for perishable commodities such as fruits and vegetables. Postharvest diseases caused by fungal pathogens can lead to significant economic losses, reduced shelf life, and potential health risks. Among the various fungal species that affect stored agricultural products, the genus *Penicillium* is one of the most prevalent and problematic (Bano et al. 2023) *Penicillium* species, such as *P. digitatum*, are known to cause green mold, a destructive disease that affects a wide range of fruits, including citrus fruits like oranges (Kanashiro et al. 2020).

The control of postharvest fungal diseases has traditionally relied on synthetic fungicides. However, concerns over the adverse effects of these chemicals on human health and the environment, as well as the development of resistance to fungal pathogens, have prompted the search for alternative, eco-friendly approaches (McLaughlin et al. 2023). In recent years, there has been an increasing interest in using natural products, such as essential oils, as potential antifungal agents in various applications, including postharvest disease management.

Essential oils are volatile, aromatic compounds extracted from plants, typically through steam distillation or other methods (Bolouri et al. 2022). These oils are known for their diverse biological activities, including antimicrobial, antioxidant, and insecticidal properties (Mssillou et al. 2022). Several studies have explored the potential of essential oils as natural preservatives and antifungal agents for various food products (Campolina et al. 2023). One promising approach is the use of essential oil mixtures, which may exhibit synergistic effects and enhanced antimicrobial activity compared to individual oils (Chouhan et al. 2017). The combination of different essential oils with complementary modes of action can potentially overcome the limitations of single oils, such as a limited spectrum of activity or rapid volatilization (Cimino et al. 2021). Among the various essential oils studied for their antifungal properties, oregano (*Origanum vulgare*), rosemary (*Rosmarinus officinalis*), and mint (*Mentha rotundifolia*.) have shown promising results against a range of fungal pathogens (Abdi-Moghadam et al. 2023). Oregano oil, rich in compounds like carvacrol and thymol, has been reported to exhibit strong antifungal activity against several *Penicillium* species, including *P. digitatum* (Moussa et al. 2021). Rosemary oil, containing compounds like α-pinene and 1,8-cineole, has also demonstrated antifungal effects against various fungi (Shahina et al. 2022). Mint oils, particularly peppermint, and spearmint, have shown inhibitory effects on the growth and sporulation of several fungal species (Kadoglidou and Chatzopoulou 2023).

In addition to their antimicrobial properties, essential oils have also been studied for their potential antioxidant activities, which can contribute to their effectiveness as preservatives (Mutlu-Ingok et al. 2020). Antioxidants play a crucial role in preventing oxidative deterioration and extending the shelf life of food products by scavenging free radicals and inhibiting lipid peroxidation (Costa et al. 2021). While the antimicrobial and antioxidant properties of essential oils have been extensively studied, their potential cytotoxic and genotoxic effects are also important considerations for their safe and effective use (Pezantes-Orellana et al. 2024). Evaluating the cytotoxicity and genotoxicity of essential oils and their mixtures is crucial to ensure their safe application in food preservation and agricultural practices (Wang et al. 2023). Various techniques have been employed to assess the antimicrobial activity of essential oils, including disc diffusion assays, broth microdilution methods, and in vivo studies (Hossain 2024). In vitro assays provide valuable insights into the antimicrobial potential of essential oils, while in vivo studies offer a more comprehensive understanding of their efficacy under real-world conditions (El-Shiekh et al. 2024).

Furthermore, the chemical composition of essential oils can vary depending on factors such as plant variety, geographical location, and extraction methods (Liñán-Atero et al. 2024). Therefore, identifying the active compounds responsible for the observed biological activities is crucial for optimizing their use and understanding their modes of action (Faizan et al. 2024).

In the context of postharvest disease management, essential oils have been explored as potential alternatives to synthetic fungicides for controlling fungal infections in various fruits and vegetables (Peralta-Ruiz et al. 2023). Several studies have investigated the efficacy of essential oils against specific fungal pathogens, such as *P. digitatum*, which cause green mold in citrus fruits (Ipinza-Concha et al. 2024).

To address the challenges posed by postharvest fungal diseases and explore eco-friendly alternatives, this study aimed to investigate the antifungal and antioxidant potential of essential oil mixtures derived from oregano, rosemary, and mint against the predominant fungal pathogen *P. digitatum* isolated from infected orange fruits. Additionally, the study aimed to optimize these essential oil mixtures for enhanced efficacy, assess their cytotoxic and genotoxic effects, and evaluate their in vivo antifungal activity against green mold caused by *P. digitatum* on orange fruits.

## Materials and methods

### Sampling and collection site

Twenty infected orange fruits were collected from four markets in Cairo, Egypt with an additional twenty healthy oranges were later obtained for pathogenicity testing after fungal isolation. Figure S1. Each sample was carefully placed in separate sterile polythene bags, labeled, and transported to the Biology lab, New Programs Administration, Faculty of Agriculture, Ain Shams University for comprehensive fungal analysis.

### Collection of plant essential oils

All essential oils including of mint (*Mentha rotundifolia*), rosemary (*Salvia Rosmarinus*), and oregano (*Origanum vulgare*) were obtained from the National Research Center (NRC), Cairo, Egypt.

### Isolation of deteriorative fungi

The fungal isolation process began by sterilizing infected citrus fruits using 70% alcohol-soaked cotton wool. Fruits were cut into small 1cm segments using a sterilized scalpel and aseptically placed these segments onto Oxytetracycline agar plates. The inoculated plates were incubated at 25 °C for 5–7 days. During incubation, multiple fungal colonies emerged, displaying diverse colorations including grey, black, green, and white. These varied colors indicated the presence of different fungal species. All emerging green fungal colonies were sub-cultured to obtain pure, uncontaminated fungal isolates for further investigation (Tian et al. 2022).

### Phenotypic identification of *Penicillium* sp. isolate

The collected isolates were identified according to their cultural and cell morphological properties using Slide cultures. The green colonies with a brush-like conidiophore structure with chains of spherical conidia were selected (Al Mohaini et al. 2022).

### Genotypic analysis of *Penicillium sp*. isolate

The most predominant *Penicillium* P15 fungal colony was shipped to the Molecular Biology Research Unit at Assiut University in order to extract DNA using a Patho-gene-spin DNA/RNA extraction kit from Intron Biotechnology Company, Korea, and perform molecular identification using 18S rRNA sequencing. Subsequently, fungal DNA sample were sent to Macrogen Company located in South Korea, for PCR and rRNA gene sequencing. PCR of the chosen isolate was conducted using ITS1 (forward) and ITS4 (reverse) primers: ITS1: (5′-TCC GTA GGT GAA CCT GCG G-3′) and ITS4: (5′-TCC TCC GCT TAT TGA TAT GC-3′). The reaction mixture included ddNTPs, and the resulting purified recombinant product was sequenced using the respective primers. Phylogenetic tree construction and sequence alignment were performed using phylogenetic relationships interferred through the neighbor-joining method using MEGA 11 software (available from https://www.megasoftware.net/) and the Cluster W algorithm (Abd-Elhalim et al. 2023).

### Standard inoculum

The culture of *Penicillium* P15 isolate was streaked onto Potato Dextrose Agar (PDA) plates and incubated at 25–30 °C for 5–7 days for sporulation. Once sporulation has occurred, a sterile saline solution (0.85% NaCl) is added to the plates, allowing the spores to be suspended in the solution. The suspension is then transferred to a sterile container. The suspension is adjusted to an optical density (OD) of 0.09–0.13 at a wavelength of 530 nm using a spectrophotometer, indicating a concentration of approximately 10^6^ spore/mL (Tian et al. 2022).

### Antimicrobial activity and minimal inhibitory concentration (MIC) for the PEOs

For antimicrobial activity, the agar well diffusion method was applied. Briefly, 500 µl of the tested *Penicillium* P15 isolate (10^6^ spore/mL) was streaked on the surface of Mueller Hinton Agar (MHA) plates. Using a 6 mm diameter cork porer, wells were made on the Petri dishes filled with 8 different PEOs (Thyme, grape seed, oregano, clove, black seed, mint, cinnamon, and rosemary) and then incubated at 25 °C for 5–7 days. After this incubation period, the Inhibition zone diameter (cm) was measured (Farouk et al. 2023). All trials were carried out in triplicates.

### Mixture design for the optimization of PEOs mixture against *Penicillium P15 isolate* NPA 2024 inhibition

The levels of each combination constitution were determined using the RSM mixture design. The mathematical expression for the sum of all mixtures acts as.

1. 0.000 ≤ A: Mint ≤ 0.660,
2. 0.000 ≤ B: Oregano ≤ 0.660,
3. 0.000 ≤ C: Rosemary ≤ 0.660,

A + B + C = 1.000, in which this relationship is called the fundamental consulate of mixtures. The ideal design for optimizing process was displayed in Table S1 consisting of 16 runs. The design of the 3D triangle called for the three diluted essential oils to be placed in the combination of essential oils, with equal quantities of each component situated at the triangle’s vertices. In order to identify pure error and compare it with lack of fit, run trial Nos. 5, 13, 16, and 14 were replicated twice. Responses were expressed as a function of independent variables using cubic and quadratic models, which depended on the mixture design approach, where Y: reaction expressed in centimetres (Inhibition zone diameter). The linear term coefficients are denoted by α1, α2, and α3. The coefficients of the binary terms are denoted by α12, α22, and α23. α123 denoted the coefficient of the ternary term (Gamal et al. 2020). The most efficient mixture was used for more further studies, All trials were carried out in triplicates.

### Cytotoxicity of PEOs mixture against normal HSF cell line

Using the MTT(3-(4,5-Dimethylthiazol-2-yl)-2,5-Diphenyltetrazolium bromide) test, the biocompatibility and safety of the active ingredients in the most efficient POE combination were examined at Nawah-Scientific in Cairo, Egypt. Penicillin (100 units/ mL), streptomycin (100 mg/ mL), and 10% fetal bovine serum were added to Dulbecco’s Modified Eagle medium (DMEM) for the growth of liver cell lines. Ten concentrations of MOL that had been serially diluted twice were made. Confluent liver cell monolayers were cultivated in a 96-well microtiter plate for 24 h in order to perform the MTT test. Each well received 20 µl of 5 mg MTT after 72 h, and they were then incubated at 37 °C. After removing the medium, 150 µl of MTT solvent was applied. The cells were then incubated on an orbital shaker for fifteen minutes after the plate was covered with tin foil. In a micro-plate reader, the optical density (OD) was detected at 570 nm. Using the information gathered from subjecting the liver cell lines to various doses of the POE combination, a dose distribution curve was created (Amr et al. 2023).

### Antioxidative activity by scavenging of DPPH

The capacity of the most efficient PEOs mixture to scavenge the DPPH (1,1-diphenyl-2-picrylhydrazyl) radical was determined according to (Gargouri et al. 2019). Using the DPPH technique, 100 µL of newly made DPPH solution (0.2 µM in methanol) was combined with 100 µL of the most efficient PEOs mixture (1.5–50 mg/100 mL) and incubated for 30 min in the dark. Next, at 517 nm, the mixture’s absorbance was measured. The following formula was used to determine the percentage inhibition (%) of the DPPH radical’s scavenging capacity:

$$\:\left(\text{\%}\:\text{i}\text{n}\text{h}\text{i}\text{b}\text{i}\text{t}\text{i}\text{o}\text{n}\right)=\:\frac{Abs\:control-Abs\:sample}{Abs\:control}\:X\:100$$

### Evaluation of chromosomal aberrations in bone marrow cells of albino mice

Twenty male albino mice (25 g ± 2) were acclimatized for one week before being orally administered the most efficient PEOs mixture (100 µg/mL) for 15 consecutive days. Mice were injected intraperitoneally with colchicine (0.4 mg/kg) 2 h before sacrifice. The pellet was resuspended in a hypotonic KCl solution (0.56 g/100 mL distilled water) at 40 °C for 30 min after the femoral bone marrow had been flushed with 0.9% NaCl and centrifuged for 10 min at 1000 rpm. The cells were then centrifuged and fixed at least three times in a solution of methanol and glacial acetic acid (3:1 v/v). The cell solution was spread out onto cold slides, flame-dried, and then stained for 30 min with 10% phosphate-buffered Giemsa (pH 6.8). At least 50 well-spread metaphases per animal were microscopically analyzed (100x magnification) for structural aberrations and numerical changes, following the modified protocol of (Norizadeh Tazehkand et al. 2016).

### Gas chromatography Mass Spectrometry for the PEOs mixture

The various chemically active components in the examined essential oils were discovered using gas chromatography Mass Spectrometry (GC/MS). Essential oils were dissolved in methanol solvent. The chemical components were examined using GC/MS in conjunction with mass spectrometry. A capillary column was employed to separate the complex mixture. The column temperature was initially held at 50 °C, then increased at a steady rate up to 250 °C, held briefly, then ramped up further to 300 °C. Helium carrier gas transported the vaporized samples through the column. An auto-injector introduced small sample volumes into a heated inlet, where they were vaporized for analysis. The instrument detected the separated chemicals as they eluted from the column, collecting mass spectra. These spectra were compared to library databases to identify the specific compounds based on fragmentation patterns unique to their structures. Operating parameters for the temperature, flow rate, injection volume, etc., were methodically chosen and controlled. Thus, the system and methodology enabled the characterization of the essential oil constituents (Tian et al. 2022).

### In vivo antifungal assay

An antifungal assay was conducted using citrus fruits obtained from a local market in Cairo, Egypt. The fruits were selected based on their healthy appearance, consistent size (between 72 and 84 g), uniform color (citrus color index ranging from 3.6 to 4.5), and absence of bruises or disease. Fruits were chosen from recent harvests, reflecting the typical peak citrus harvest season in Egypt, generally from November to March. Only fruits with uniform ripening stages were included to reduce variability in their biochemical composition, ensuring the consistency of the assay. Egyptian market conditions, including ambient temperature and humidity, as well as storage and transport methods, were considered when selecting fruits to ensure they were in a condition reflective of typical consumer access Before being wound, the selected fruits were cleaned by soaking them in a solution of 1% sodium hypochlorite for 2 min, rinsing them with tap water to get rid of any leftover disinfectant, and allowing them to air dry in a sterile area. To prepare for the assay, a uniform wound (2 mm deep and 4 mm in diameter) was created on the equatorial side of each fruit using a sterile puncher. Subsequently, 20 µL of a mixture of plant essential oils (PEOs) at various concentrations (0%, 0.5%, and 1%) was injected into each wound. Thirty minutes later, each wound was reinjected with 20 µL of a *P. digitatum* spore suspension (10^6^ spores/mL). The fruits treated with PEOs and the control group were arranged in plastic boxes with a polyethylene lining, measuring 26 cm × 35 cm, and kept at 25 °C for incubation. Using a vernier caliper, the lesions’ diameters were measured 3, 5, 7, and 10 days after injection (Chen et al. 2016, 2019). All trials are carried out in triplicates. The inhibition in diameter (%) was calculated as follows.

$$ \begin{gathered} {\text{Inhibition}}\;{\text{over}}\;{\text{control }}(\% ) = \left( {{\text{Lesion}}\;{\text{diameter }}({\text{Control}})} \right. \hfill \\ \quad \quad \quad \quad \quad \quad \quad \quad \quad \quad \quad \quad \left. { - {\text{Lesion}}\;{\text{diameter}}\;({\text{treatment}})} \right)/{\text{Lesion}} \hfill \\ \quad \quad \quad \quad \quad \quad \quad \quad \quad \quad \quad \quad {\text{diameter}}({\text{Control}}) \times 100 \hfill \\ \end{gathered} $$

### Statistical analysis

The outcome data were analyzed using one-way ANOVA integrating “Design Expert” software (Version 12, StatــEase Inc; Minneapolis, USA) at a level of statistical significance of 0.05.

## Results

### Isolation of deteriorative fungi

**Fig. 1:**
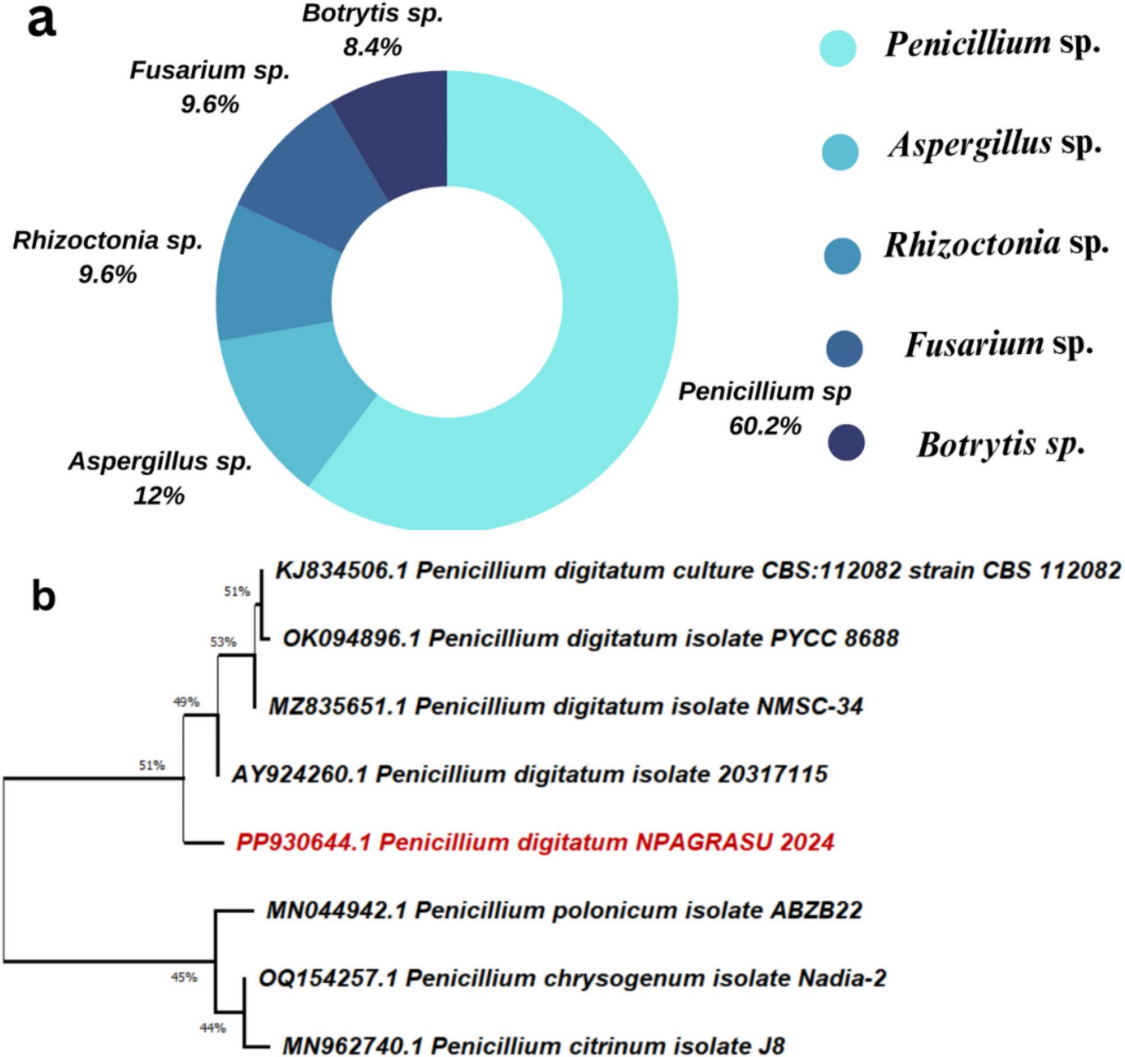
**a** Abundance percentage (%) of obtained fungal isolates collected from infected orange fruits. **b** Phylogenetic analysis of *Penicillium digitaum* NPAGRASU 2024 in comparison to seven *Penicillium* strains in GenBank based on 16 S rRNA gene sequences

Figure 1a presents data on the abundance of different fungal species isolated from infected orange fruits. The data show the percentage distribution of each fungal species identified. The results indicate that *Penicillium* sp. is the predominant fungal species, accounting for 60.2% of the total isolates. This suggests that *Penicillium* sp. is the most prevalent and abundant fungus affecting the orange fruits in the study. The second most abundant fungal genus is *Aspergillus* sp., representing 12% of the isolates. *Rhizoctonia* sp. and *Fusarium* sp. both have a lower abundance, each contributing 9.6% of the total isolates. *Botrytis *sp., a fungus known to cause gray mold, has the least abundance among the identified fungi, accounting for only 8.4% of the isolates. These results reveal that *Penicillium sp*. is the primary fungal pathogen responsible for the majority of the infections observed in the orange fruits studied. *Penicillium* P15 isolate was the predominant isolate. Therefore, it was picked up and subcultured for further studies.

### Phenotypic identification of *Penicillium* P15 isolate

The fungi species represented in supplementary data Figure S2 exhibit diverse morphological features. *Penicillium* sp. displays a brush-like conidiophore structure with chains of spherical conidia.

### Molecular identification of *Penicillium P15 isolate*

Molecular identification of the P15 *Penicillium* isolate, which was the most abundant isolate obtained, was performed using 18S rRNA gene sequence analysis and depicted in Fig. 1b. The 18S rRNA gene was amplified and sequenced, and the resulting sequence was used for phylogenetic analysis and comparison with reference sequences in the GenBank database. The phylogenetic tree was constructed using the MEGA 11 software, employing the Neighbor-Joining method and the Cluster W algorithm. The phylogenetic analysis revealed that the P15 isolates clustered closely with the *P. digitatum* strain, indicating a high degree (9 8%) of similarity. The *P15* isolate was identified as *P. digitatum* NPAGRASU 2024, with a 18S rRNA gene sequence deposited in the GenBank database under the accession number PP930644.1. Within the phylogenetic tree, the *P. digitatum* NPAGRASU isolate (PP930644.1) formed a distinct lineage separate from other closely related *Penicillium* strains, such as KJ834506.1 *P. digitatum* culture CBS:112,082, OK094936.1 *P. digitatum* isolate PYCC 8688, MZ835651.1 *P. digitatum* isolate NMSC-34, and AY924260.1 *P. digitatum* isolate 20,317,115.

### Antifungal activity of PEOs against *Penicillium digitatum*

Supplementary data in Figure S3 illustrates the inhibition zone diameters (IZD) in millimeters for oregano, rosemary, and mint essential oils against the most abundant *Penicillium digitatum*. Oregano oil exhibited the highest antifungal activity, with a maximum IZD of 4.2 cm. Rosemary oil also demonstrated potent antifungal effects, with IZD values ranging from 2.3 to 2.8 cm. Mint oil showed comparatively lower antibacterial potential, with the highest IZD of 1.3 cm. The larger inhibition zone diameters for oregano and rosemary oils indicate their superior ability to inhibit bacterial growth compared to mint oil in this assay.

### Optimization of essential oil mixtures against *Penicillium digitatum* using mixture design of response surface methodology

The provided data in Table 1 represents a mixture design experiment employing l-optimal mixture design of the response surface methodology (RSM) to optimize the concentrations of three essential oils (oregano, mint, and rosemary) against the fungus *Penicillium digitatum*. The table compares the actual and predicted values for the Inhibition Zone Diameter (IZD) and the percentage of growth reduction. The results indicate a good fit between the actual and predicted values, suggesting the RSM model effectively captures the relationship between the essential oil concentrations and their antifungal activity. Notably, Run 8 exhibited the highest actual percentage reduction (99.65%), making it the most effective run against the *Penicilliu digitatum*. Several other runs, including 5, 12, 13, 14, and 16, also demonstrated highly promising antifungal activity, with actual percentage reductions greater than or equal to 98%. Overall, the study highlights the potential of optimized essential oil mixtures as effective antifungal agents against *Penicillium digitatum*.

**Table 1:** Actual and predicted IZD (cm) and reduction growth (%) of the *Penicillium digitatum* using the PEOs mixture

Run	Oregano (mL)	Rosemary (mL)	Mint (mL)	Actual IZD	Predicted IZD	Actual % reduction	Predicted % reduction
1	0.065568	0.66	0.274432	0.12	0.1161	74.00	74.15
2	0.66	0.32	0.02	4.9	5.00	86.00	87.84
3	0.66	0.119282	0.220718	4.3	4.19	83.00	80.93
4	0.101408	0.456706	0.441886	2.5	2.47	67.00	66.40
5	0.316769	0.323015	0.360216	5.6	5.32	98.99	98.68
6	0	0.34	0.66	0.8	0.7684	65.00	62.84
7	0.251225	0.66	0.0887746	3.1	3.19	72.50	74.13
8	0.462578	0.537422	0	6.1	5.98	99.65	97.52
9	0.065568	0.66	0.274432	0.15	0.1161	75.00	74.15
10	0.56	0	0.44	4.5	4.57	85.00	86.36
11	0	0.34	0.66	0.7	0.7684	60.00	62.84
12	0.367899	0	0.632101	5.2	5.18	98.90	98.51
13	0.316769	0.323015	0.360216	5.1	5.32	98.00	98.68
14	0.316769	0.323015	0.360216	5.1	5.32	98.00	98.68
15	0.205636	0.19755	0.596814	3.2	3.15	70.00	69.14
16	0.316769	0.323015	0.360216	5.4	5.32	98.50	98.68

### Mixtures against *Penicillium digitatum * using response surface methodology

The supplementary data in Table S2 present the analysis of variance (ANOVA) results for the inhibition zone diameter (IZD) and percentage reduction in growth for the mixture design experiment involving three essential oils (oregano, mint, and rosemary) and their interactions against the *Penicillium digitatum* strain.

For IZD, the model and linear mixture terms are highly significant (p-value < 0.0001), indicating that the concentrations of the essential oils have a significant impact on the IZD. The interaction term AB (p-value = 0.0195) is also significant, suggesting a synergistic effect between two of the essential oils on the IZD. The lack-of-fit is insignificant (p-value = 0.4814), implying that the model adequately fits the data. The model has a high R-squared value of 0.9915 and an adjusted R-squared of 0.9872, indicating a good fit. The coefficient of variation (C.V. %) is 5.03%, and the mean IZD is 2.93 cm.

For the percentage reduction in growth, the model and linear mixture terms are highly significant (p-value < 0.0001), indicating that the concentrations of the essential oils significantly affect the growth reduction. The interaction terms AB (p-value = 0.0082) and AC (p-value = 0.0198) are also significant, suggesting synergistic effects between certain essential oil pairs on growth reduction. The lack-of-fit is insignificant (p-value = 0.4418), implying that the model adequately fits the data. The model has a high R-squared value of 0.9866 and an adjusted R-squared of 0.9799. The coefficient of variation (C.V. %) is 6.05%, and the mean percentage reduction in growth is 57.56%.

**Fig. 2:**
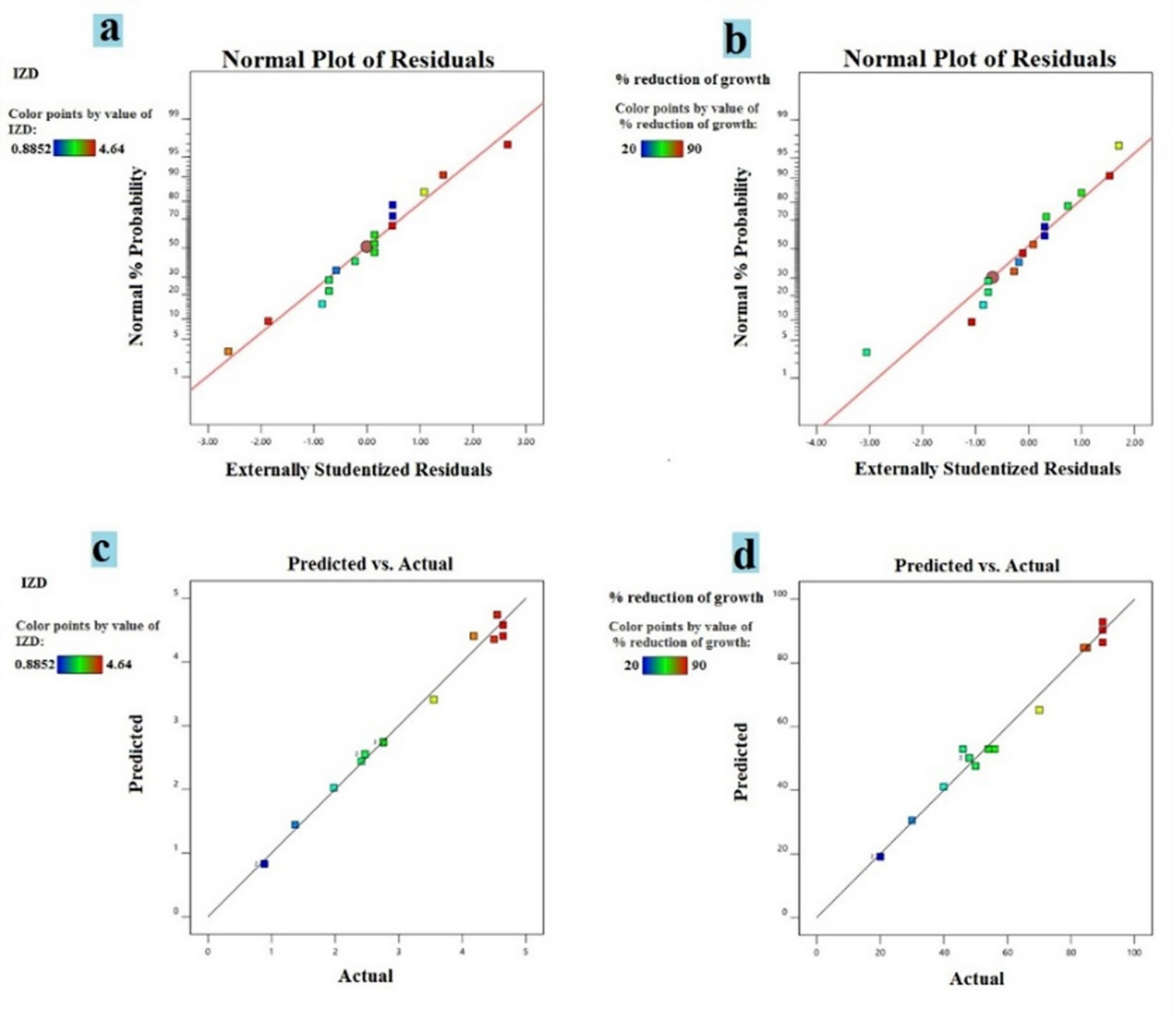
Ternary Contour Plots and Response Surface Plots for Optimizing Essential Oil Mixtures against *Penicillium digitatum*. **a** Normal plot for IZD, **b**Normal plot for growth reduction (%), **c** predicted against actual IZD plot, **d** predicted against actual growth reduction (%)

The images provided in Fig. 2 appear to be diagnostic plots related to the ANOVA analysis. Image (a) shows a normal probability plot of residuals for the inhibition zone diameter (IZD) response, while image (b) shows the normal probability plot of residuals for the percentage reduction in growth response.

**Fig. 3:**
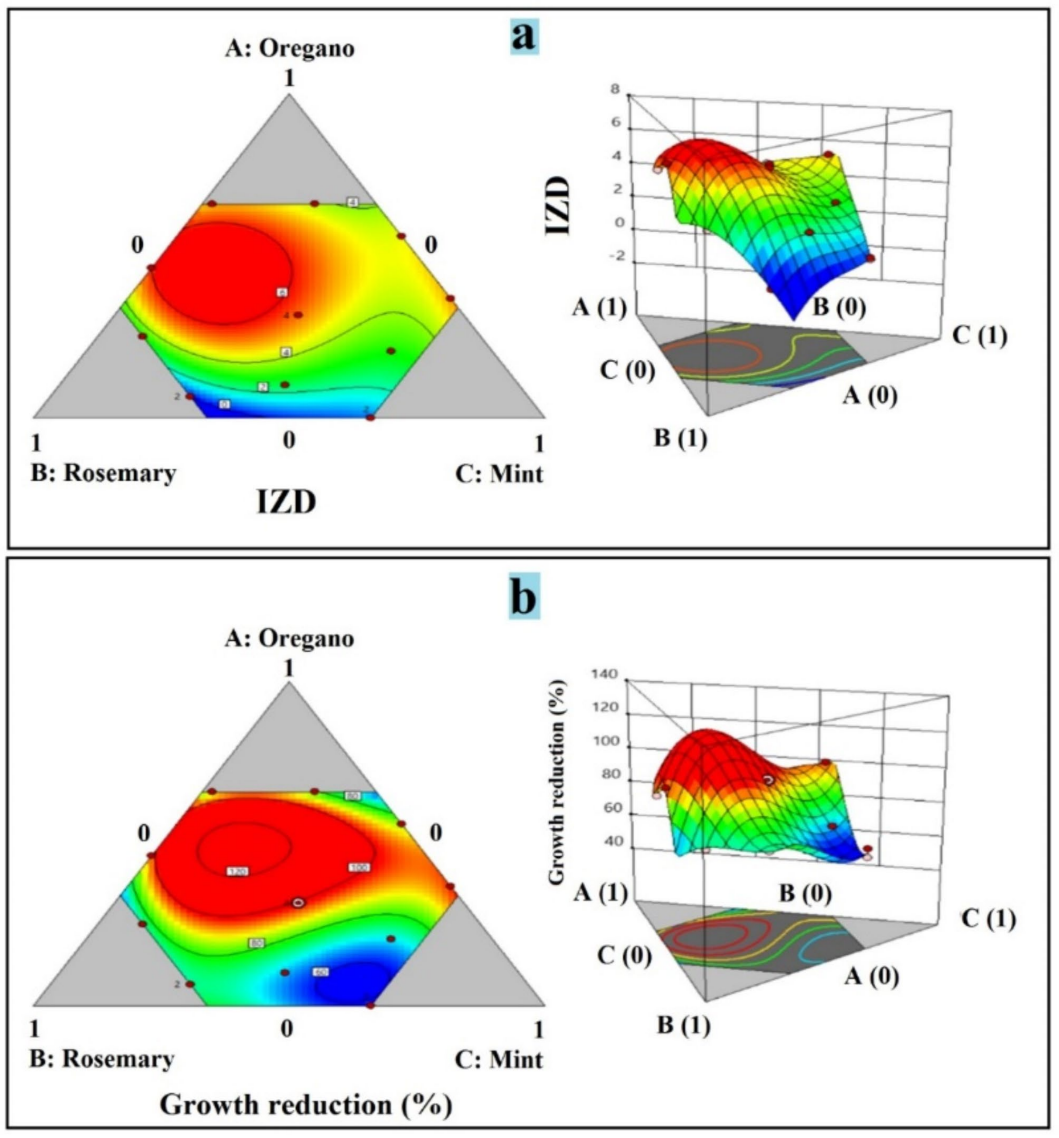
2D and 3D response surface plots for optimizing essential oil mixtures against *Penicillium digitatum*. **a** interaction between Oregano, rosemary and mint against IZD (cm) of *P. digitatum*. **b** interaction between Oregano, rosemary and mint against growth reduction (%) of *P. digitatum*

Images (c) and (d) are predicted vs. actual value plots for IZD and percentage reduction in growth, respectively. These plots help assess the adequacy of the fitted model by comparing the predicted values from the model against the actual observed values. The normal probability plot of residuals should approximate a straight line, indicating that the residuals are normally distributed, which is a key assumption of ANOVA. The predicted compared to actual value plots should show points randomly scattered around the diagonal line, suggesting that the model adequately predicts the responses across the range of values. Figure 3 represents ternary contour plots and 3D surface plots generated from the mixture design experiment involving oregano, rosemary, and mint essential oils, indicating the inhibition zone diameter (IZD) and percentage reduction in growth (%) against *Penicillium digitatum* strain. The ternary contour plots (a, b, and c) show the regions of different response values (indicated by the color gradient) within the feasible composition space defined by the three essential oil components. The 3D surface plots (right side of each image) offer a clearer visualization of the response surfaces, allowing for the identification of optimal composition regions that maximize the desired responses.

### Antioxidant activity

**Fig. 4:**
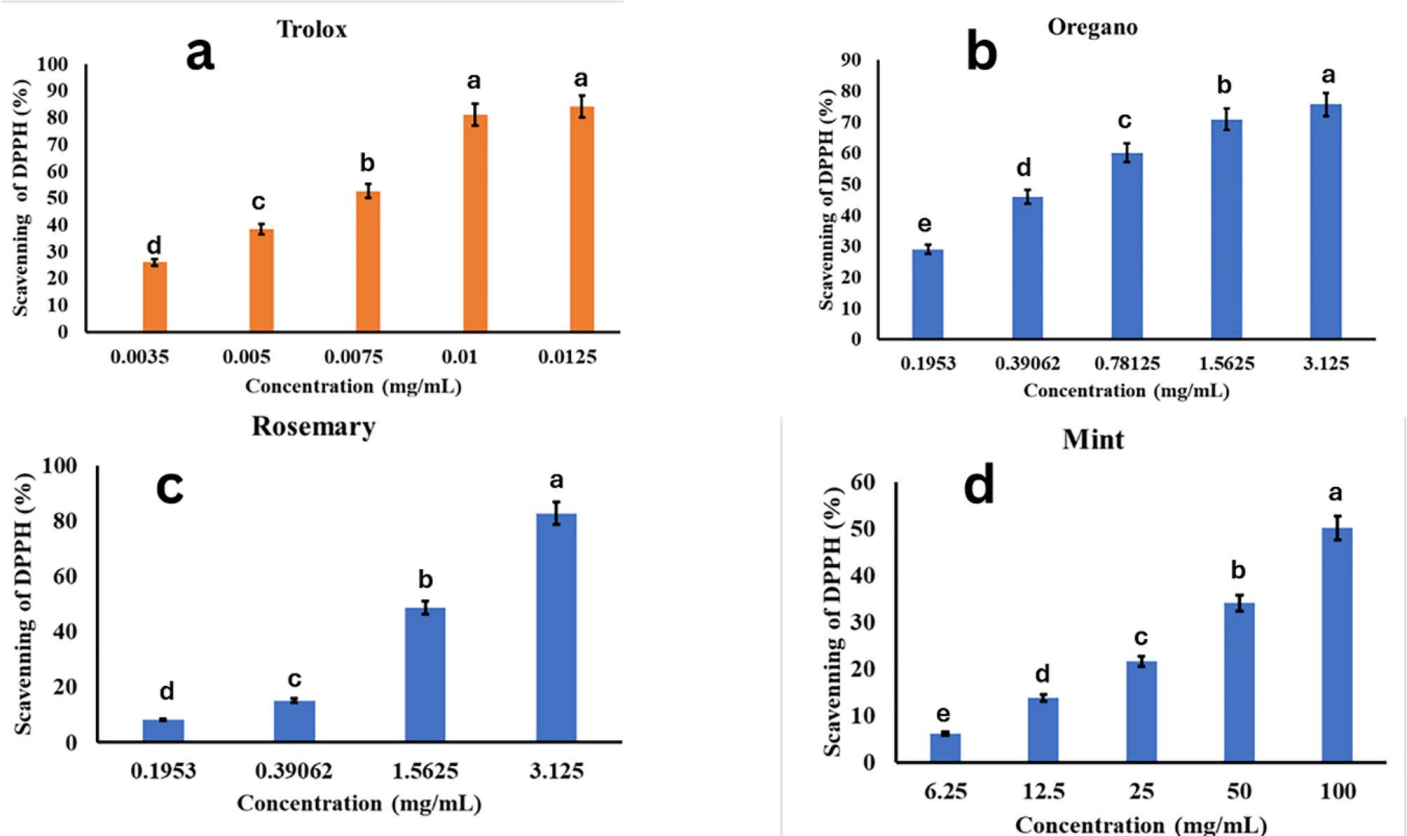
DPPH radical scavenging activity (%) of three essential oils compared to the standard antioxidant Trolox. **a** Trolox. **b** oregano, **c** rosemary, **d** mint

Based on the data in Fig. 4, Trolox, as a positive control, exhibited the highest scavenging activity across the tested concentration range, reaching around 83% at 0.0125 mg/mL. Among the essential oils, oregano showed the highest scavenging activity, reaching approximately 75% at 3.125 mg/mL. It outperformed rosemary and mint at most of the tested concentrations, indicating its superior antioxidant potential. Rosemary demonstrated moderate antioxidant activity, with a maximum scavenging activity of around 75% at 3.125 mg/mL, similar to oregano at the same concentration. However, at lower concentrations, rosemary exhibited lower scavenging activity compared to oregano. Mint exhibited the lowest scavenging activity among the three essential oils, with a maximum of approximately 53% at 100 mg/mL, which is a relatively high concentration compared to the other oils. It is noteworthy that while Trolox achieved high scavenging activity at low concentrations (e.g., 83% at 0.0125 mg/mL), the essential oils required significantly higher concentrations to reach comparable levels of activity. This is likely due to the complex composition of the essential oils and the presence of various other compounds besides the antioxidant constituents.

Overall, the results indicate that oregano possessed the strongest antioxidant activity among the three essential oils tested, followed by rosemary and then mint. However, all three essential oils exhibited lower antioxidant potency compared to the synthetic antioxidant Trolox when considering their effective concentration ranges.

### Cytotoxicity potential of PEOs mixture against liver cell line

**Fig. 5:**
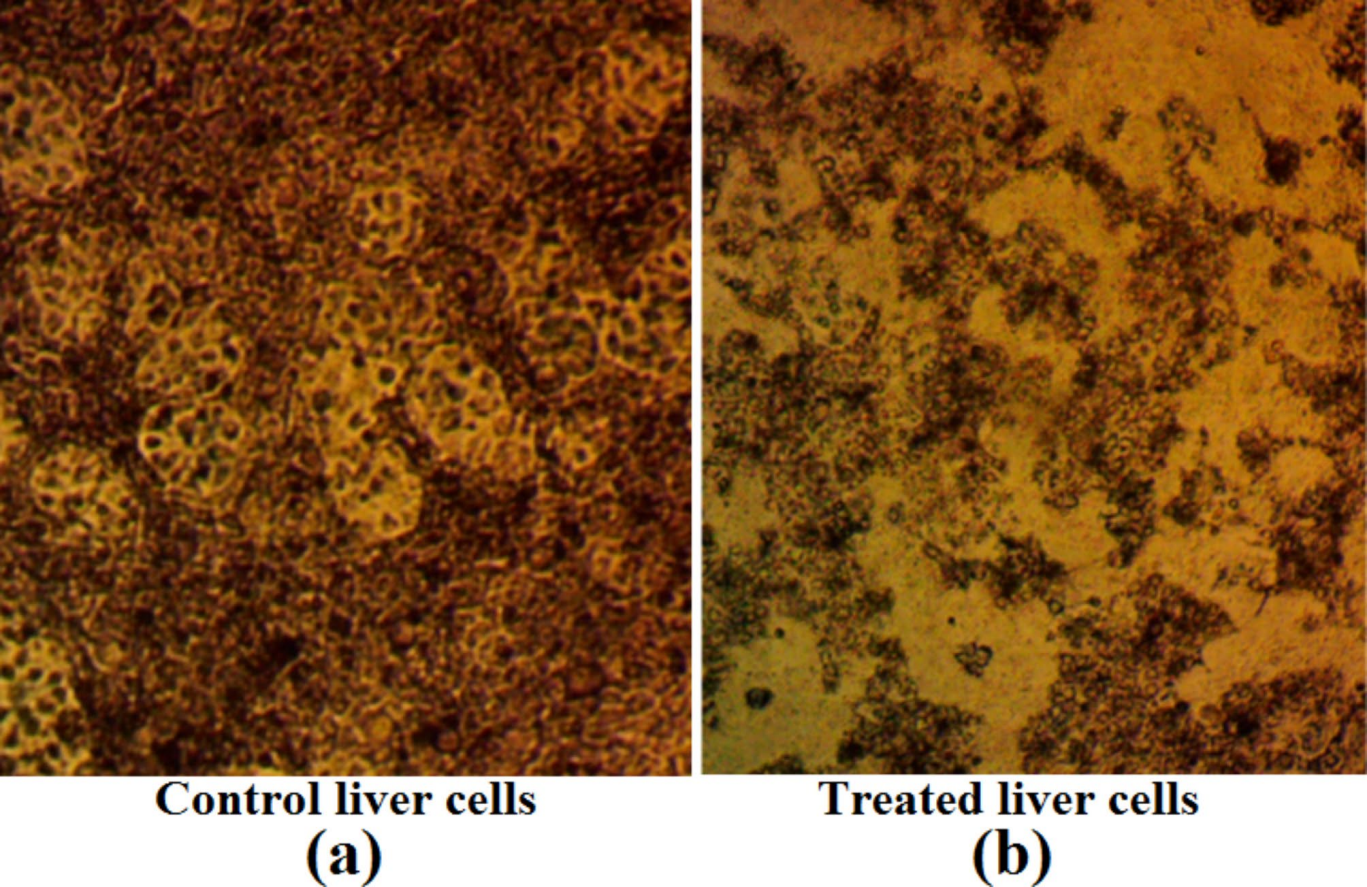
Evaluation of cytotoxic effects of PEOs mixture on liver cell line Using MTT Assay. **a** control cells exhibiting a typical, evenly distributed morphology with defined shapes, visible nuclei, and cytoplasmic structures. **b** treated liver cells with 300 µg/mL PEOs mixture illustrating significant morphological changes such as reduced cell density, and altered cell shapes (rounded or shrunken). Magnification power 100X

The study depicted in Fig. 5 evaluated the cytotoxic effects of the PEOs mixture on a liver cell line using the MTT assay. Image a shows the control cells exhibiting a typical, evenly distributed morphology with defined shapes, visible nuclei, and cytoplasmic structures. In contrast, image b depicts significant morphological changes in the cells treated with the PEOs mixture at a concentration of 300 µg/mL. These changes include reduced cell density, altered cell shapes (rounded or shrunken), and the presence of dark spots or debris indicative of cellular damage or death. The observed morphological abnormalities in the treated sample suggest that the PEOs induced cytotoxic effects on the liver cell line, as assessed by the MTT assay, which measures cell viability and metabolic activity.

### Cytogenetic analysis of PEOs mixture

**Fig. 6:**
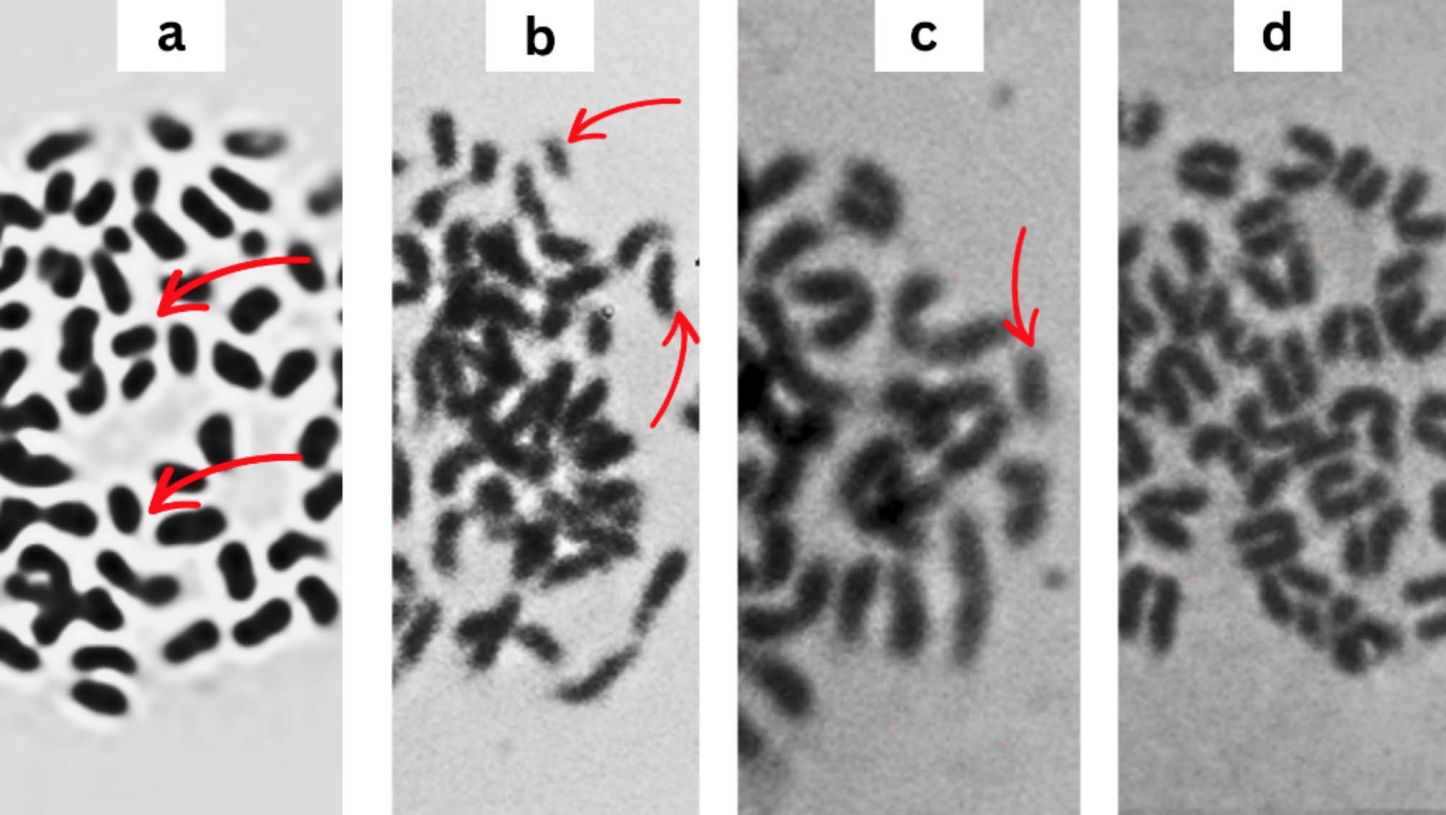
Metaphase spreads from the bone marrow of mice fed on EOs, **a** Centromeric attenuation, **b** Deletion, **c** Fragmentation, **d** Normal

The supplementary data in Table S3 and Fig. 6 present data on various types of chromosomal aberrations observed under three treatment conditions: Control, MMC (mitomycin C), and PEOs mixture (essential oils mixture). Compared to the control, the EOs mixture treatment did not induce any significant increase in structural chromosomal aberrations such as deletions, fragments, or centromeric attenuations. The total number of structural aberrations was similar between the control (0.4 ± 0.24) and PEOs mixture (0.2 ± 0.1999) treatments. Furthermore, the PEOs mixture did not have a substantial impact on numerical chromosomal aberrations, with a total numerical aberration rate of 1 ± 0.6323, which was only slightly higher than the control (0.6 ± 0.39). The mitotic index, which represents the percentage of cells undergoing mitosis, was also comparable between the control (80) and EOs mixture (73.5) treatments. Overall, the data suggests that the EOs mixture did not exhibit significant genotoxic effects and its impact on chromosomal integrity was similar to the control condition.

### Histological analysis of PEOs mixture effect on different mice organs

**Fig. 7:**
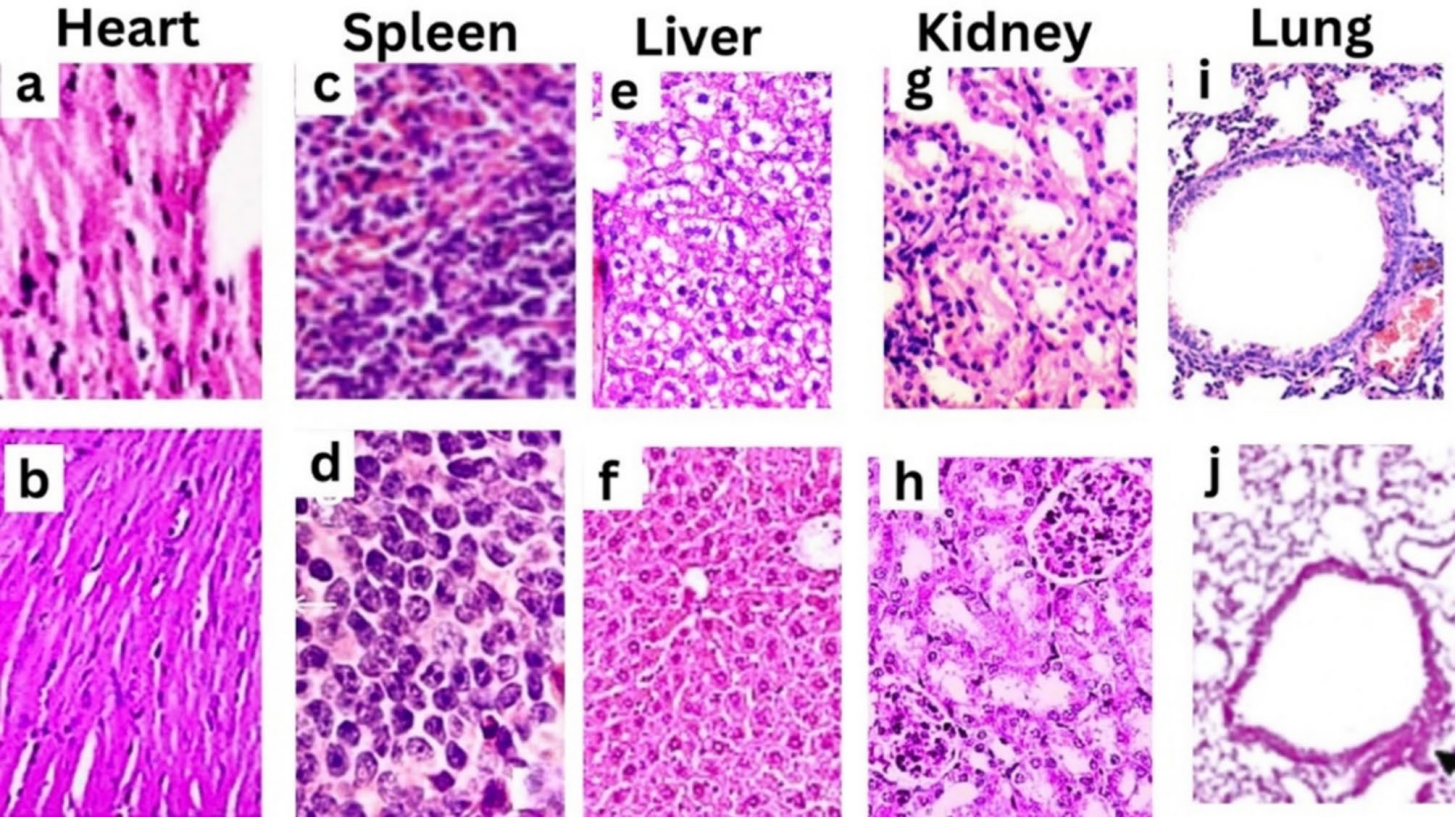
Histological analysis of PEOs mixture effect on different mice organs. All images were recorded using magnification power of 100 X. **a**, **c**, **e**, **g** and **i** are for control treatments. **b**, **d**, **f**, **h**, and **j** are treated mice with 1% of PEOs mixture concentration

Histological results in Fig. 7 illustrated that the myocardial muscle tissue section showed intra-muscular edema with mild congestion of intermuscular blood capillaries. The degree of myocardial muscle damage revealed [(1) Mild-focal myocyte damage or small multifocal degeneration]. Lung tissue sections demonstrated typical histological lung construction, with folded columnar bronchiole epithelial cells, normal fibrous tissue distribution, normal vasculature with sparse perivascular connective tissue, and fine, delicate inter-alveolar septa separating the airspaces. With a narrow inter-alveolar septa score of 0, the alveoli seemed swollen. Sections of splenic tissue had lengthy, irregular, or many rounds of lymphoid cells arranged in a periarteriolar lymphoid sheath that floated inside the crimson pulp, displaying a distinct and noticeable edge grade (0). The kidney tissue slice had a typical histological structure with circumscribing glomeruli, Bowman’s capsule, and capillary tufts. The renal tubules exhibited normal organization and an undamaged epithelial lining in both the proximal and distal convoluted tubules (scoring 0). Liver tissue sections revealed ballooning degeneration of hepatocytes and disorganization of hepatic cords. Hyperplasia of Kupffer cells and narrowing of hepatic sinusoids were also noticed in grade (II).

### Gas chromatography Mass Spectrometry (GC/MS) analysis of the PEOs mixture

Table 2 illustrates the chemical composition of the three oils. The GC-MS analysis of the essential oils revealed the main chemical constituents and their relative abundances. For oregano oil, the primary compound was carvacrol, representing 83.25% of the total area at a retention time (RT) of 13.42 min. Other notable compounds included o-cymene (6.34%, RT 6.30 min), γ-terpinene (5.22%, RT 7.20 min), and thymol (4.64%, RT 13.18 min). Mint oil was dominated by levomenthol, accounting for 38.67% of the total area at RT 10.03 min, followed by trans-cyclohexanone, 5-methyl-2-(1-methyl ethyl)- (24.36%, RT 9.28 min), and a minor presence of D-limonene (0.59%, RT 6.51 min). In rosemary oil, eucalyptol was the most abundant compound, contributing 16.53% at RT 6.45 min, with levomenthol (9.23%, RT 10.02 min), caryophyllene (2.05%, RT 16.47 min), camphene (1.85%, RT 4.79 min), and endo-borneol (2.34%, RT 9.70 min) as additional constituents. These results highlight the distinct chemical profiles of the oils, reflecting their unique compositions and potential bioactive properties.

**Table 2:** The chemical composition of the three oils (oregano, rosemary, and mint) using GC/MS analysis

Retention time (RT)	Compound name	Area %	Molecular formula
Oregano oil			
6.30	o-Cymene	6.34	C_10_H_14_
7.20	ç-Terpinene	5.22	C_10_H_16_
13.18	Thymol	4.64	C_10_H_14_O
13.42	Cavarcol	83.25	C_10_H_14_O
Mint oil			
9.28 min	Cyclohexanone, 5-methyl-2-(1-methyl ethyl)-, trans-	24.36%	C_10_H_18_O
10.03 min	Levomenthol	38.67%	C_1_0H_20_O
6.51 min	D-Limonene	0.59%	C_10_H_16_
Rosemary oil			
16.47	Caryophyllene	2.05	C_15_H_24_
6.45	Eucalyptol	16.53	C_10_H_18_O
4.79	Camphene	1.85	C_10_H_16_
10.02	Levomenthol	9.23	C_10_H_20_O
9.70	Endo-Borneol	2.34	C_10_H_18_O

### In vivo antifungal activities of EOs mixture against *P. digitatum*

**Fig. 8:**
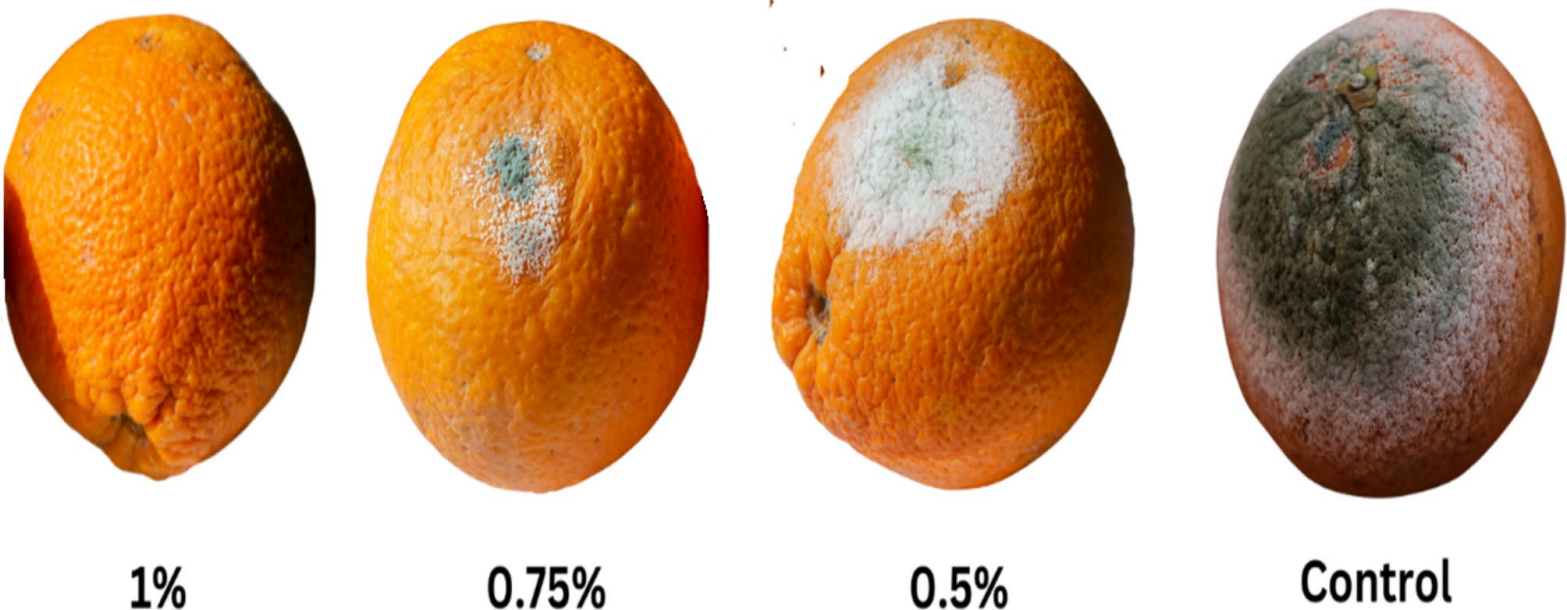
Control efficacy of PEOs mixture at concentrations from 0.5 to 1% against green mold caused by *P. digitatum*

The data depicted in supplementary Table S4 and Fig. 8 show oranges treated with different concentrations (0.5%, 0.75%, and 1%) of an essential oils (EOs) mixture, along with an untreated control orange. The data in the table provides information on the lesion diameter (in mm) and the percentage inhibition of the green mold caused by *P. digitatum* at different time points (3, 5, and 7 days post-inoculation) for the same EOs mixture concentrations. Compared to the control treatment, which showed a progressive increase in lesion diameter from 25.3 ± 1.15 mm on day 3 to 60.8 ± 1.64 mm on day 7, the EOs mixture treatments exhibited a significant reduction in lesion diameter. At 0.5% concentration, the lesion diameters were 10.6 ± 1.58 mm, 15.8 ± 1.08 mm, and 30.5 ± 1.39 mm on days 3, 5, and 7, respectively. The 1% concentration showed even greater inhibition, with lesion diameters of 7.8 ± 1.54 mm, 11.7 ± 1.55 mm, and 24.2 ± 1.66 mm on the respective days. The percentage inhibition of the green mold, relative to the control, increased with higher concentrations of the EOs mixture. On day 7, the 0.5% concentration showed 49 ± 0.75% inhibition, while the 1% concentration exhibited 60 ± 0.37% inhibition. The visual representation in the image demonstrates the antifungal activity of the EOs mixture against *P. digitatum*, with the treated oranges showing significantly less mold growth and lesion development compared to the untreated control orange.

## Discussion

The present study aimed to explore the potential of essential oil mixtures derived from oregano, rosemary, and mint as effective antifungal and antioxidant agents against *P. digitatum*, the predominant fungal pathogen responsible for green mold in orange fruits. The findings of this research contribute to the development of eco-friendly strategies for postharvest disease management and extend our understanding of the biological activities of these essential oils and their mixtures.

The initial step in this study involved the isolation and identification of the predominant fungal species responsible for the deterioration of orange fruits. The results revealed that *Penicillium sp*. was the most abundant fungal genus, accounting for 60.2% of the total isolates Fig. 1. This finding is consistent with previous reports indicating that *Penicillium* species, particularly *P. digitatum*, are among the primary causative agents of green mold in citrus fruits (Wang et al. 2024). The isolate identified as *P. digitatum* NPAGRASU 2024 was selected for further studies based on its prevalence and pathogenicity.

The antimicrobial activity of the individual essential oils (oregano, rosemary, and mint) was initially evaluated against the *P. digitatum* isolate using the disc diffusion method. Oregano oil exhibited the highest antifungal activity, with a maximum inhibition zone diameter (IZD) of 4.2 cm, followed by rosemary oil (IZD range of 2.3–2.8 cm) and mint oil (IZD of 1.3 cm) as shown in Figure S3. These findings are in agreement with previous studies that have reported the potent antifungal properties of oregano and rosemary oils against various fungal pathogens [Citlali Colín-Chavez, Kuljinder Kaur]. To enhance the antifungal efficacy and explore potential synergistic effects, a mixture design experiment was conducted using L-optimal mixture design of response surface methodology (RSM). The optimization process resulted in several promising mixtures, with Run 8 (46.26% oregano, 53.74% rosemary) exhibiting the highest actual percentage reduction (99.65%) against *P. digitatum* Table 1. This synergistic effect of the essential oil mixture is consistent with previous reports demonstrating that combining multiple essential oils can lead to enhanced antimicrobial activity due to their complementary modes of action (Chen et al. 2022). The ANOVA results in Table S1 further validated the significance of the model and the linear mixture terms, as well as the interaction terms AB (oregano × rosemary) and AC (oregano × mint), indicating the importance of these interactions in determining the antifungal activity of the mixtures. The diagnostic plots in Fig. 2 confirmed the adequacy of the fitted model, with the normal probability plots of residuals approximating a straight line and the predicted vs. actual value plots showing a random scatter around the diagonal line.

In addition to their antimicrobial properties, the antioxidant activities of the essential oils were evaluated using the DPPH radical scavenging assay. Oregano oil exhibited the highest scavenging activity, reaching approximately 75% at 3.125 mg/mL, followed by rosemary oil with a similar maximum activity of around 75% at the same concentration as shown in Fig. 4. These findings align with previous studies that have reported the potent antioxidant properties of oregano and rosemary oils, attributable to their phenolic compounds, such as carvacrol and rosmarinic acid, respectively (Et-tazy et al. 2023). The antioxidant activity of essential oils can contribute to their effectiveness as preservatives, as they can scavenge free radicals and inhibit lipid peroxidation, thereby extending the shelf life of food products (Campolina et al. 2023). However, it is important to note that while Trolox achieved high scavenging activity at low concentrations (e.g., 83% at 0.0125 mg/mL), the essential oils required significantly higher concentrations to reach comparable levels of activity, likely due to their complex composition and the presence of various other compounds besides the antioxidant constituents.

The potential cytotoxic and genotoxic effects of the optimized essential oil mixture were evaluated to ensure its safe application. The MTT assay revealed significant morphological changes and reduced cell viability in liver cells treated with the three-oil mixture at 200 µg/mL as shown in Fig. 5, indicating cytotoxic effects at this concentration. However, it is important to note that the effective antifungal concentrations of the optimized mixture were lower (e.g., Run 8 with 99.65% reduction at a combined concentration of approximately 46% oregano and 54% rosemary), suggesting that the cytotoxic effects may be minimized at the concentrations required for antifungal activity.

The chromosomal aberration analysis in Table S2 and Fig. 6 demonstrated that the essential oil mixture did not induce significant structural or numerical chromosomal aberrations compared to the control treatment. The total number of structural aberrations, total numerical aberrations, and the mitotic index were comparable between the control and the essential oil mixture treatment, suggesting a lack of genotoxic effects.

These findings are consistent with previous studies that have reported the relatively low cytotoxicity and genotoxicity of essential oils at concentrations below their effective antimicrobial or antioxidant levels (Chen et al. 2023). However, it is crucial to consider the specific composition and concentrations of the essential oils, as well as the target application, when assessing their potential toxicity (Brah et al. 2023).

The optimized essential oil mixture (PEOs) was further evaluated for its in vivo antifungal activity against green mold caused by *P. digitatum* on orange fruits. The results in Table S3 and Fig. 8 demonstrated a significant reduction in lesion diameter and inhibition of mold growth in the treated oranges compared to the untreated control. At a concentration of 1% (v/v), the PEOs mixture exhibited a 60 ± 0.37% inhibition of green mold on day 7, indicating its effectiveness as an antifungal agent against *P. digitatum* in a real-world setting. These findings are consistent with previous studies that have explored the use of essential oils for postharvest disease control in various fruits and vegetables (Elhelaly et al. 2022). The antifungal activity of essential oils can be attributed to their ability to disrupt the cell membrane and interfere with various cellular processes in fungal pathogens, such as respiration, ion transport, and enzyme activities (Tian et al. 2022).

To assess the potential toxicity of the essential oil mixture on various organs, a histological analysis was conducted on mice treated with the optimized PEOs mixture. The results of Fig. 7 revealed no significant pathological changes in the lung, spleen, and kidney tissues of the treated mice compared to the control group. However, mild focal myocyte damage and ballooning degeneration of hepatocytes were observed in the heart and liver tissues, respectively, indicating potential toxicity at the tested concentration (1% PEOs mixture).

These findings highlight the importance of determining the appropriate concentrations and exposure levels of essential oils to minimize potential adverse effects while maintaining their desired biological activities. Similar observations of organ-specific toxicity have been reported in previous studies evaluating the in vivo effects of essential oils and their constituents (Ibrahim et al. 2018).

The chemical compositions of the individual essential oils (oregano, rosemary, and mint) were analyzed using gas chromatography-mass spectrometry (GC-MS) as shown in Table 2. The major compounds identified in oregano oil were caryophyllene, eucalyptol, camphene, levomenthol, and borneol. In mint oil, the main components were cyclohexanone, 5-methyl-2-(1-methyl ethyl)-, trans- and levomenthol, while rosemary oil was rich in o-cymene, 5-isopropyl-2-methylphenol, thymol, and γ-terpinene. The presence of these compounds aligns with previous reports on the chemical compositions of these essential oils (Ninich et al. 2024). The identified compounds, such as carvacrol, thymol, and eucalyptol, have been associated with various biological activities, including antimicrobial, antioxidant, and anti-inflammatory effects (Gupta et al. 2024). However, it is important to note that the biological activities of essential oils are often attributed to the synergistic effects of their multiple constituents rather than the activity of a single compound (Benamar-Aissa et al. 2024). The chemical composition analysis provides valuable insights into the potential active components responsible for the observed antifungal and antioxidant properties of the essential oil mixtures. The present study aimed to explore the potential of essential oil mixtures derived from oregano, rosemary, and mint as effective antifungal and antioxidant agents against *P. digitatum*, the predominant fungal pathogen responsible for green mold in orange fruits. The findings of this research contribute to the development of eco-friendly strategies for postharvest disease management and extend our understanding of the biological activities of these essential oils and their mixtures.

In conclusion, this study explored the potential of essential oil mixtures derived from oregano, rosemary, and mint as effective antifungal and antioxidant agents against *P. digitatum*, the predominant fungal pathogen responsible for green mold in orange fruits. The findings demonstrated the promising antifungal activity of the optimized essential oil mixture, with significant inhibition of mold growth and lesion development in oranges treated with the mixture. The mixture also exhibited potent antioxidant properties, contributing to its potential as a natural preservative. Importantly, the cytotoxicity and genotoxicity assessments revealed minimal genotoxic effects of the optimized mixture, although potential cytotoxicity was observed at higher concentrations. The histological analysis highlighted the need for careful determination of appropriate concentrations and exposure levels to minimize potential adverse effects on specific organs. Overall, this study contributes to the development of eco-friendly strategies for postharvest disease management and extends our understanding of the biological activities of essential oil mixtures. The findings pave the way for further research and potential applications in the agricultural and food industries, promoting sustainable practices and reducing reliance on synthetic fungicides.

## Electronic Supplementary Material

Below is the link to the electronic supplementary material.

Supplementary Material 1

## Data Availability

The raw data and analyzed data used during the current study are available from the corresponding author upon reasonable request. *P. digitatum* was isolated from infected orange fruits and identified then deposited in GenBank with gene accession number PP930644 https://www.ncbi.nlm.nih.gov/nuccore/PP930644. It was deposited in Egypt Microbial Culture Collection, MIRCEN, Cairo, Egypt with number EMCC 358874. https://www.10.12210/ccinfo.EMCC.
