# Supplementary material for: Optimization, characterization and biosafety of oregano, rosemary and mint oil mixture against Penicillium digitatum in citrus using L-optimal mixture design
Source: AMB Express. 2025 Jan 27;15:14. doi: 10.1186/s13568-024-01806-0 (PMC11772652; doi:10.1186/s13568-024-01806-0)
Supplement: Supplementary file 1 — Supplementary Material 1 [file 13568_2024_1806_MOESM1_ESM.pdf]

**Optimization, characterization and biosafety of Oregano oil mixture against *Penicillium digitatum* in citrus**

**Rahaf Khaled[1], Sara Mohamed[1], Amira Mohamed[1], Aya Khairy[1], Hesham El-hariry[2],Ashraf Bakry[3], Neima K. Elsenosy[3], Naglaa Ebeed[3], Salem S. Salem[4], Thanaa ElNoby[5], Samah H. Abu-Hussien[6]***

[1]Biotechnology Program, New Programs Administration, Faculty of Agriculture, Ain Shams University, P.O. Box 68, Cairo, 11241, Egypt

[2]Food Science department, Faculty of Agriculture, Ain Shams University, P.O. Box 68, Cairo, 11241, Egypt

[3]Genetics department, Faculty of Agriculture, Ain Shams University, P.O. Box 68, Cairo, 11241, Egypt

[4]Department of Botany and Microbiology, Faculty of Science, Al-Azhar University, Nasr City, Cairo, Egypt

[5]Agriculture Economics department, Faculty of Agriculture, Ain Shams University, P.O. Box 68, Cairo, 11241, Egypt

[6]Agriculture Microbiology Department, Faculty of Agriculture, Ain Shams University, P.O. Box 68, Cairo, 11241, Egypt

**Correspondence: Samah H. Abu-Hussien (samah_hashem1@agr.asu.edu.eg)**

**Table (S1)** Matrix for the mixture design for PEOs mixture using mixture design

| Run | Oregano (mL) | Rosemary (mL) | Mint (mL) |
|---|---|---|---|
| 1 | 0.065568 | 0.66 | 0.274432 |
| 2 | 0.66 | 0.32 | 0.02 |
| 3 | 0.66 | 0.119282 | 0.220718 |
| 4 | 0.101408 | 0.456706 | 0.441886 |
| 5 | 0.316769 | 0.323015 | 0.360216 |
| 6 | 0 | 0.34 | 0.66 |
| 7 | 0.251225 | 0.66 | 0.0887746 |
| 8 | 0.462578 | 0.537422 | 0 |
| 9 | 0.065568 | 0.66 | 0.274432 |
| 10 | 0.56 | 0 | 0.44 |
| 11 | 0 | 0.34 | 0.66 |
| 12 | 0.367899 | 0 | 0.632101 |
| 13 | 0.316769 | 0.323015 | 0.360216 |
| 14 | 0.316769 | 0.323015 | 0.360216 |
| 15 | 0.205636 | 0.19755 | 0.596814 |
| 16 | 0.316769 | 0.323015 | 0.360216 |

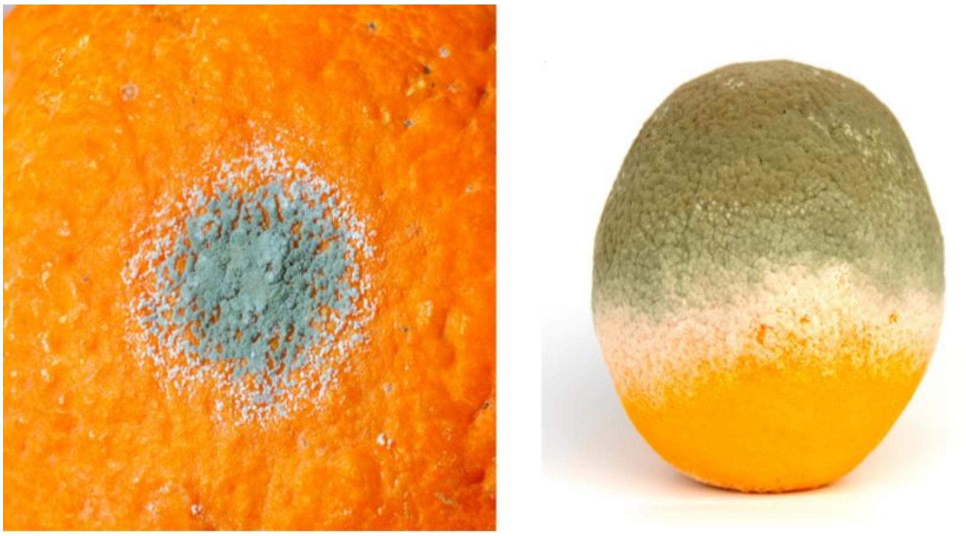

**Figure S1:** Infected orange fruits by green mold.

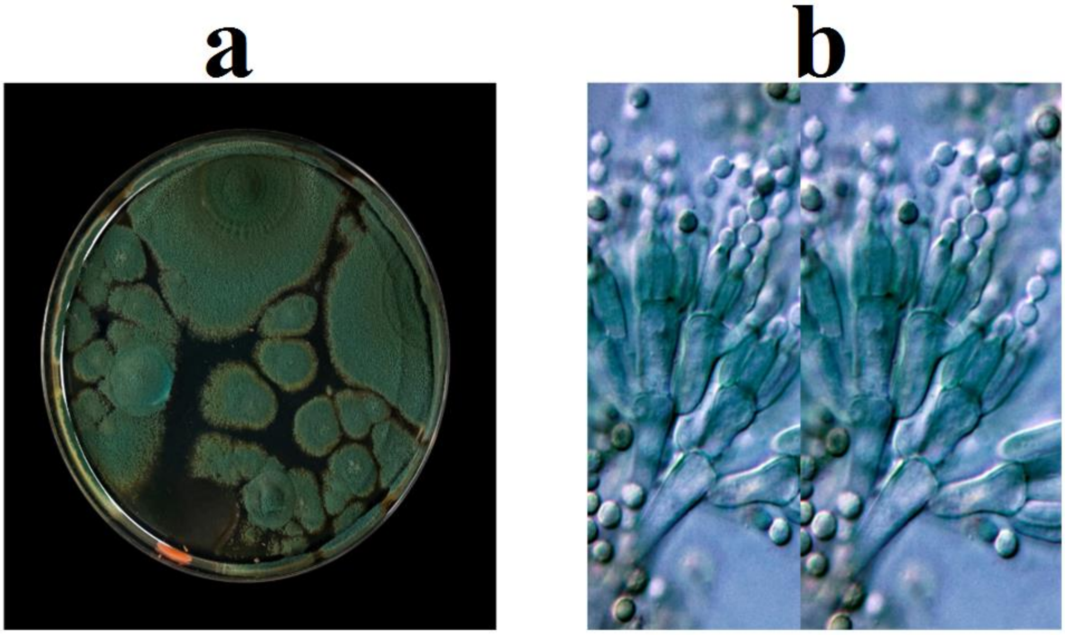


**Figure S2:** Colony characteristics (a) and microscopic examination (b) of the selected *Penicillium P15 isolate* using light microscopy with a magnification power of 40X**.**

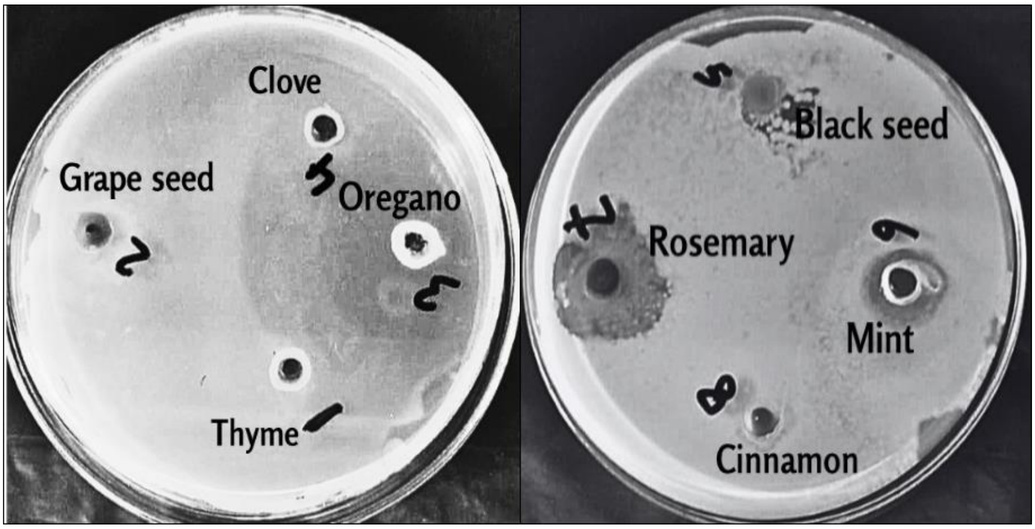


**Figure S3:** Antimicrobial potential of the 9 plant essential oils (Thyme, grape seed, oregano, clove, black seed, mint, cinnamon, and rosemary) against *Penicillium P15 isolate* NPA 2024 isolate indicating the highest IZD for oregano, rosemary, and mint.

**Table (S2):** Analysis of variance (ANOVA) for the IZD (cm) and reduction growth (%) against *Penicillium P15 isolate* NPA 2024 isolates using the PEOs mixture.

| **IZD (cm)** | **Source** | **Sum of Squares** | **df** | **Mean Square** | **F-value** | **p-value** | |
|---|---|---|---|---|---|---|---|
| | **Model** | 25.15 | 5 | 5.03 | 232.48 | < 0.0001 | significant |
| | [(1)]Linear Mixture | 24.80 | 2 | 12.40 | 573.24 | < 0.0001 | |
| | AB | 0.1670 | 1 | 0.1670 | 7.72 | 0.0195 | |
| | AC | 0.0916 | 1 | 0.0916 | 4.23 | 0.0667 | |
| | BC | 0.0429 | 1 | 0.0429 | 1.98 | 0.1893 | |
| | **Residual** | 0.2163 | 10 | 0.0216 | | | |
| | Lack of Fit | 0.1105 | 5 | 0.0221 | 1.04 | 0.4814 | not significant |
| **Reduction growth (%)** | **Std. Dev.** 0.1471 | **$R^2$** 0.9915 | **Adjusted $R^2$** 0.9872 | | **Mean** | 2.93 | **C.V. %** 5.03 |
| | **Source** | **Sum of Squares** | **df** | **Mean Square** | **F-value** | **p-value** | |
| | **Model** | 8920.60 | 5 | 1784.12 | 147.04 | < 0.0001 | significant |
| | [(1)]Linear Mixture | 8721.99 | 2 | 4361.00 | 359.41 | < 0.0001 | |
| | AB | 131.02 | 1 | 131.02 | 10.80 | 0.0082 | |

<table>
<tr><td rowspan="6"></td><td colspan="2">AC</td><td colspan="2">93.13</td><td colspan="2">1</td><td>93.13</td><td>7.67</td><td>0.0198</td><td colspan="2"></td></tr>
<tr><td colspan="2">BC</td><td colspan="2">16.99</td><td colspan="2">1</td><td>16.99</td><td>1.40</td><td>0.2640</td><td colspan="2"></td></tr>
<tr><td colspan="2">Residual</td><td colspan="2">121.34</td><td colspan="2">10</td><td>12.13</td><td></td><td></td><td colspan="2"></td></tr>
<tr><td colspan="2">Lack of Fit</td><td colspan="2">64.84</td><td colspan="2">5</td><td>12.97</td><td>1.15</td><td>0.4418</td><td colspan="2">not significant</td></tr>
<tr><td>Std. Dev.</td><td>3.48</td><td>R²</td><td>0.9866</td><td>Adjusted R²</td><td>0.9799</td><td></td><td>Mean</td><td>57.56</td><td>C.V. %</td><td>6.05</td></tr>
</table>

**Table (S3):** Evaluation of the genotoxic potential of an essential oils mixture through chromosomal aberration analysis

| Types | Treatments | | |
|---|---|---|---|
| | Control | MMC | EOs mixture |
| Deletion | 0.4 ± 0.24 | 11.2 ± 1.82 | 0.4 ± 0.24 |
| Fragments | 0.0 ± 0.0 | 1.6 ± 0.39 | 0.0 ± 0.0 |
| Centromeric attenuations | 0.0 ± 0.0 | 5.8 ± 1.35 | 0.0 ± 0.0 |
| T. structural aberrations | 0.4 ± 0.24 | ***18.6 ± 1.69 | 0.2 ± 0.1999 |
| 2n | 49.4 ± 0.39 | 33 ± 1.22 | 49 ± 0.6323 |
| > 2n | 0.4 ± 0.39 | 3.2 ± 0.86 | 1 ± 0.6323 |
| < 2n | 0.2 ± 0.19 | 9.6 ± 2.29 | 0.0 ± 0.0 |
| T. numerical aberrations | 0.6 ± 0.39 | 17 ± 1.22 | 1 ± 0.6323 |
| No. examed cells | 10000 | 10000 | 10000 |
| Mitotic Index | 80 | 11.2 ± 1.82 | 73.5 |

**Note:** The mean difference is significant at the .05 level.

**Table (S4)** Lesion diameter and inhibition diameter (%) after 7 days of incubation at 25 °C.

| (dpi).dex | Days Post inoculation (DPI) | Concentration of PEOs (%,v/v) | | |
|---|---|---|---|---|
| | | 0.0 (control) | 0.5 | 1 |
| Lesion diameter (LD) | 3 | 25.3±1.15$^{a}$ | 10.6±1.58$^{b}$ | 7.8±1.54$^{c}$ |
| | 5 | 38.8±1.72$^{a}$ | 15.8±1.08$^{b}$ | 11.7±1.55$^{c}$ |
| | 7 | 60.8±1.64$^{a}$ | 30.5±1.39$^{b}$ | 24.2±1.66$^{c}$ |
| Inhibition over control (%) | 3 | | 58±1.23$^{a}$ | 69±1.15$^{b}$ |
| | 5 | | 59±0.88$^{a}$ | 69±0.28$^{b}$ |
| | 7 | | 49±0.75$^{a}$ | 60±0.37$^{b}$ |
